# High Impact = High Statistical Standards? Not Necessarily So

**DOI:** 10.1371/journal.pone.0056180

**Published:** 2013-02-13

**Authors:** Patrizio E. Tressoldi, David Giofré, Francesco Sella, Geoff Cumming

**Affiliations:** 1 Dipartimento di Psicologia Generale, Università di Padova, Padova, Italy; 2 D.P.S.S., Università di Padova, Padova, Italy; 3 School of Psychological Science, La TrobeUniversity, Victoria, Australia; Cardiff University, United Kingdom

## Abstract

What are the statistical practices of articles published in journals with a high impact factor? Are there differences compared with articles published in journals with a somewhat lower impact factor that have adopted editorial policies to reduce the impact of limitations of Null Hypothesis Significance Testing? To investigate these questions, the current study analyzed all articles related to psychological, neuropsychological and medical issues, published in 2011 in four journals with high impact factors: Science, Nature, The New England Journal of Medicine and The Lancet, and three journals with relatively lower impact factors: Neuropsychology, Journal of Experimental Psychology-Applied and the American Journal of Public Health. Results show that Null Hypothesis Significance Testing without any use of confidence intervals, effect size, prospective power and model estimation, is the prevalent statistical practice used in articles published in Nature, 89%, followed by articles published in Science, 42%. By contrast, in all other journals, both with high and lower impact factors, most articles report confidence intervals and/or effect size measures. We interpreted these differences as consequences of the editorial policies adopted by the journal editors, which are probably the most effective means to improve the statistical practices in journals with high or low impact factors.

## Introduction

Scientific papers published in journals with the highest impact factor (IF) are selected after a severe examination by peer reviews, which assess their scientific value and methodological quality. Assessing the statistical methods used is an important part of judging methodological quality. In Life and Behavioral Sciences, null hypothesis significance testing (NHST) is very often used, even though many scholars have, since the 1960s [1], identified its limited ability to answer the questions researchers ask and described damaging errors researchers commit when using it.

NHST starts by assuming that a null hypothesis, H0, is true, where H0 is typically a statement of zero effect, zero difference, or zero correlation in the population of interest. A *p* value is then calculated, where *p* is the probability, if H0 is true, of obtaining the observed result, or more extreme. A low *p* value, typically *p*<.05, throws doubt on H0 and leads to the rejection of H0 and a conclusion that the effect in question is statistically significant. Many techniques have been recommended as better than NHST, most notably for our purposes the reporting of effect sizes and confidence intervals (CIs), and the fitting of quantitative models. Statistical power is defined only in the context of NHST, but even so, we regard use of prospective statistical power—the calculation of power before collecting data, usually to guide choice of *N*—as an advance, because such use can help avoid some of the pitfalls of NHST.

Our main aim is to study how often NHST, despite its serious flaws, and alternative better methods are used in leading scientific journals, and to compare frequencies with those of journals with relatively lower impact factors that have adopted explicit editorial policies to improve statistical practices, requiring for example reporting of measures of effect size, and confidence intervals. We surveyed articles related to psychological, neuropsychological and medical issues to include a range of disciplines related to human life.

### The limitations of NHST

Cohen [2], Kline [3] and Cumming [4] provided detailed explanations of the problems of NHST, whose typical use was termed the “null ritual” by Gigerenzer*et al.* [5]. They described this as: (a) Set up a statistical null hypothesis of “no mean difference” or “zero correlation.” (b) Don't specify the predictions of your research hypothesis or of any alternative substantive hypotheses; (c) Use 5% as a convention for rejecting the null; (d) If significant, accept your research hypothesis. (e) Always perform this procedure.

For the purposes of this study, we will mention five of the limitations of NHST that seriously undermine its scientific value and consequently the reliability of results reported in studies that rely on NHST.

The first is that NHST centers on rejection of the null hypothesis, at a stated probability level, usually 0.05. Consequently, researchers can at most obtain the answer “Yes, there is a difference from zero”. However very often researchers are primarily interested in a “No” answer, and are therefore tempted to commit the logical fallacy of stating: “if H0 is rejected then H0 is false, if H0 is not rejected then H0 is true” [6]. Failing to reject H0 should usually be regarded as an open verdict—no conclusion is justified.

The second limitation is that the *p* value is very likely to be quite different if an experiment is repeated. For example if a two-tailed result gives *p* = 0.05, there is an 80% chance the one-tailed *p* value from a replication will fall in the interval (.00008, .44), a 10% chance that *p*<.00008, and fully a 10% chance that *p*>.44 [7]. In other words, a *p* value provides only extremely vague information about a result's repeatability. Researchers do not appreciate this weakness of *p* [8].

The third limitation is that the conclusion “Yes, there is a difference from zero” is almost always true. In other words, the null hypothesis is almost never exactly correct. The probability that H0 will be rejected increases with the sample size (*N*), so the result of NHST says as much, or more, about *N* as about any hypothesis. One example is that a very low two-tailed correlation coefficient *r* = 0.10 is not sufficient to reject the H0 of a zero true correlation with *p*<0.05, up to *N* = 380 participants. Above this number, H0 can be rejected.

The fourth limitation is that NHST does not give an estimate of the difference from H0, which is a measure of effect size, even when the answer is “Yes, there is a difference from zero”.

The fifth limitation is that NHST does not provide any information about precision, meaning the likely error in an estimate of a parameter, such as a mean, proportion, or correlation. Any estimate based on a physical, biological or behavioral measure will contain error, and it is fundamental to know how large this error is likely to be.

### Statistical recommendations

To reduce the impact of these five and other limitations of NHST, psychological and medical scientific associations have made statistical recommendations to be adopted by all editors and reviewers. For example, for psychology, the 6^th^ edition of the *American Psychological Association Publication Manual* [9] emphasizes the prospective estimation of statistical power “….*take seriously the statistical power considerations associated with the tests of hypotheses*” (p. 30), the use of confidence intervals (CIs) and effect size “*complete reporting of all tested hypotheses and estimates of appropriate effect sizes and confidence intervals are the minimum expectations for all APA journals*” (p. 33), and, especially : “*Wherever possible, base discussion and interpretation of results on point and interval estimates*.” (p. 34). In other words, researchers should base their conclusions on their observed effect sizes (point estimates), and the CIs (interval estimates) on those effect sizes.

For medicine, the International Committee of Medical Journal Editors (ICMJE) released the “Uniform Requirements for Manuscripts” (URM). In the Statistics paragraph of the updated April 2010 version, it is recommended “*When possible, quantify findings and present them with appropriate indicators of measurement error or uncertainty (such as confidence intervals). Avoid relying solely on statistical hypothesis testing, such as P values, which fail to convey important information about effect size*.” (p. 13).

Similarly recommendations are emphasized in the CONSORT Statement [10]:

*“For all outcomes, authors should provide a confidence interval to indicate the precision (uncertainty) of the estimate. A 95% confidence interval is conventional, but occasionally other levels are used”* and *“Although P values may be provided in addition to confidence intervals, results should not be reported solely as P values. Results should be reported for all planned primary and secondary end points, not just for analyses that were statistically significant or ‘interesting’”*. (item 17a).

How many studies published in journals with the highest IF adopt these recommendations? Are there differences with journals with lower IF in which editorial policy requires adoption of them? These are the questions addressed in the current study. To answer these questions we examined articles, and coded whether they use CIs, ESs, prospective power, and model estimation or model fitting procedures. If they used none of those four techniques, we coded whether they used NHST. We also noted whether CIs and/or ESs were interpreted, and whether CIs were shown as error bars in figures.

## Methods

### Criteria for selection of journals

Following the ISI Science and Social Science Report Index, among the journals with the highest Impact Factor (HIF) reporting behavioral, neuropsychological and medical investigations, we selected *Science*, *Nature*, *Nature Neuroscience*, *Nature Medicine*, *The Lancet* and *The New England Journal of Medicine (NEJM)*. Among the journals with relatively lower IF (LIF), and whose editorial policy requires adoption of APA or CONSORT statistical recommendations, we selected the *Journal of Experimental Psychology –Applied (JEP-A)*, *Neuropsychology* and the *American Journal of Public Health (AJPH)*. Their IFs are reported in Table S1 of Supplementary Materials. Except Science and Nature journals, all other journals state in their submission guidelines that authors are required to analyze their data according to URM or APA statistical recommendations.

The six HIF journals had impact factors between 15.5 (*Nature Neuroscience*) and 53.2 (*NEJM*), with mean of 32.8. The three LIF journals had impact factors between 2.2 (JEP-A) and 3.9 (AJPH), mean 3.3.

### Articles' inclusion criteria

To compare broadly similar studies, we restricted our survey to empirical studies with human participants related to behavioral, neuropsychological and medical investigations using quantitative and inferential statistics, published in the 2011 volumes. We excluded studies of animals and of biological or physical materials. Furthermore we did not include meta-analyses or studies carried out on single cases. Beyond these selection criteria, we did not attempt the perhaps impossible task to select subsets of articles from the different journals that used similar designs, or similar measures. Designs, measures, and other aspects of experiments, are likely to vary across disciplines and journals, and may influence choice of statistical technique. Our aim was to compare across journals, using all relevant articles, noting that many variables could contribute to any differences we found.

### Statistical practice classification

The articles passing the inclusion criteria were classified according to the following categories (see the complete scoring method in the Supplementary Material):

**Confidence Intervals:** At least one CI was reported, in a table, figure, or text.

**Effect size:** At least one measure of effect size was reported and recognized as such by authors. For example, the correlation coefficient *r* or R^2^ was reported and authors referred to it as an effect size measure.

**Effect size with confidence intervals:** At least one ES with CI was reported.

**Model estimation:** Model fitting or estimation, or model comparison was reported. This may have involved fitting a stated quantitative model to data using, for example, Bayesian methods or structural equation modeling or the assessment of goodness of fit to permit comparison of two or more models.

**Power:** Prospective statistical power was mentioned and estimated.

**NHST:** When no one of CI, ES, Model or Power estimation, was reported, but *p* values, or mention of null hypothesis or statistical significance was included.

**Interpretation of Confidence Intervals:** At least one CI was explicitly mentioned in the data interpretation or discussion.

**Effect size interpretation:** At least one effect size was explicitly mentioned in the data interpretation or discussion.

**Error bars in Figures:** The type of error bar (Standard deviation, CI, Standard Error, other (e.g. box-plots), or no error bar included, was recorded. This category was included because of the value of such figures, and to follow the survey of psychology journals by Cumming *et al.* [11], which identified a rapid increase in use of figures with error bars over 1998–2006.

First, we coded each article for ESs, CIs, Model and Power estimation. Only when none of the above practices were detected, was the article examined to determine whether it used NHST.

Note the use of a liberal approach: A practice was coded as present even if an article included only a single example of that practice.

## Results

The database is exhaustive for 2011, and so descriptive statistics could be considered sufficient. However the database may also be considered a sample from a longer time period, and so we added 95% confidence intervals [12] as an estimate of precision of our measures.

In Table S2 of Supplementary material we report the raw number of articles included for each journal. For the six HIF journals, between 5 (Nature) and 173 (NEJM) articles were included, a total of 356 articles. For the three LIF journals, between 30 (JEP-A) and 147 (AJPH) articles were included, a total of 252 articles.

### Coders' agreement

All selected *Science* and *Nature* (all journals) articles and a randomly chosen 20% of articles from the other journals using the option Random Integer Generator from the website: www.random.org, were coded independently by two of the authors. Percentage agreement was 100% for use of NHST, and ranged from 90% for Confidence Interval and Effect size interpretation, to 99% for Model estimation.

### Use of Confidence Intervals

**Figure 1:**
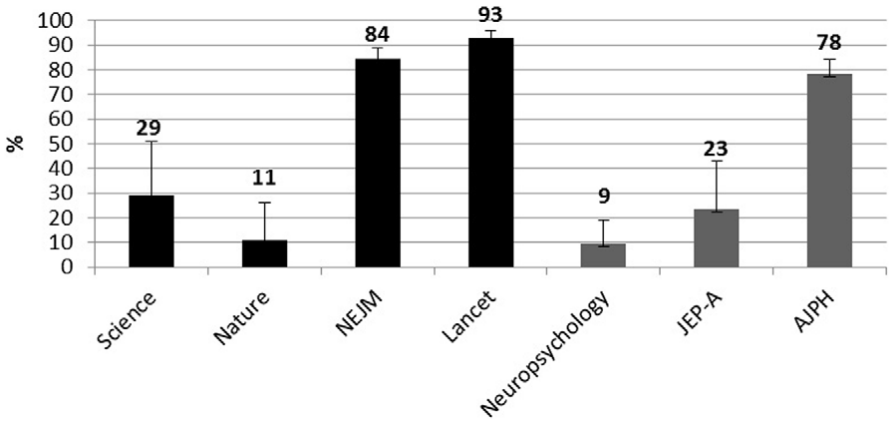
Percentages of selected articles in each journal reporting a CI. **Black histograms = HIF journals**. Gray Histograms = LIF journals. Error bars are 95% CI.

Figure 1 reports the percentage of articles that included a CI.

### Use of Effect Size

**Figure 2:**
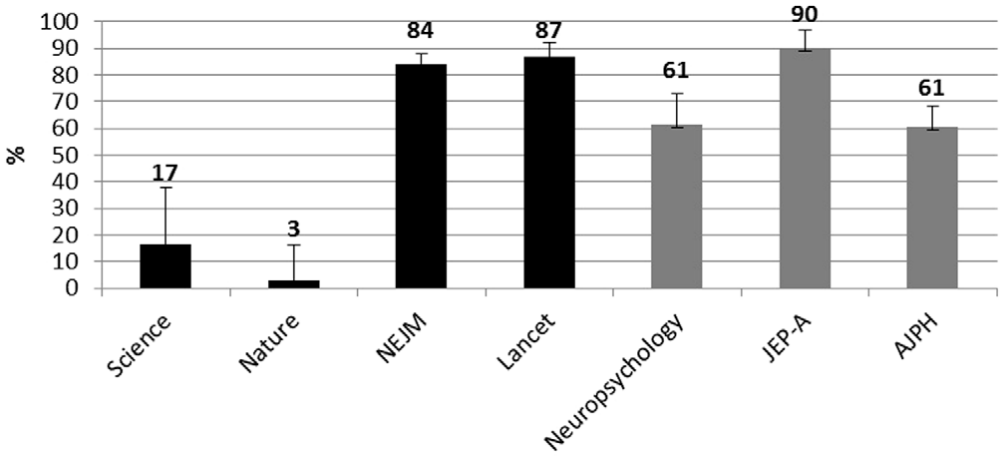
Percentages of selected articles in each journal that reported a measure stated to be an effect size. Black histograms = HIF journals. Gray Histograms = LIF journals. Error bars are 95% CI.

Figure 2 reports the percentage of articles that included a measure of ES.

**Figure 3:**
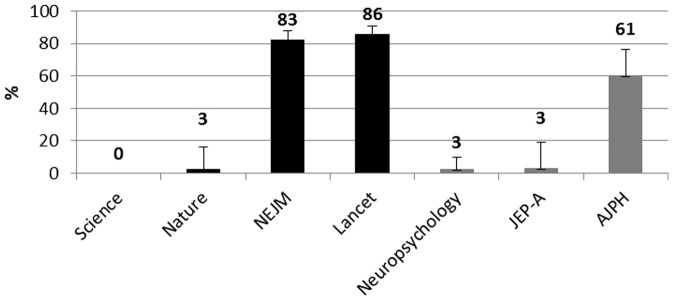
Percentages of selected articles in each journal that reported a measure of effect size with CI. Black histograms = HIF journals. Gray Histograms = LIF journals. Error bars are 95% CIs.

Figure 3, reports the percentage of articles that included a measure of ES with CI.

### Model estimation

**Figure 4:**
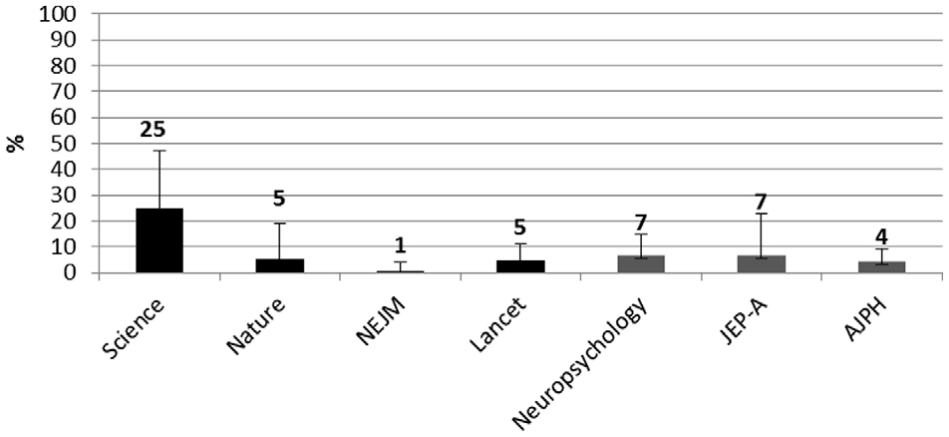
Percentages of selected articles in each journal that reported model estimation. Black histograms = HIF journals. Gray Histograms = LIF journals. Error bars are 95% CI.

Data related to the use of model estimation are reported in Figure 4. This practice was used in 25% of articles in Science. It was used in 7% of articles in Neuropsychology and JEP-A, and in 5% or less in the remaining journals.

### Use of prospective Power

**Figure 5:**
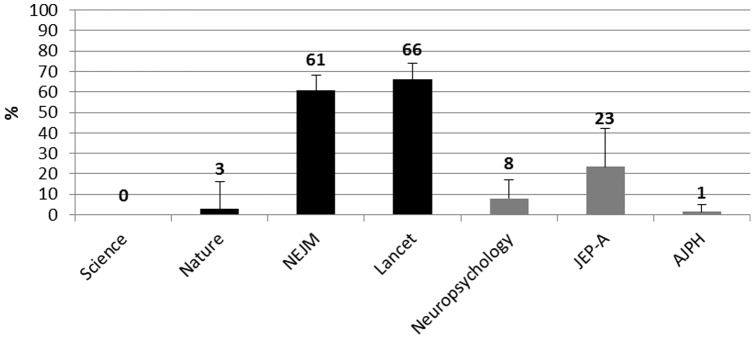
Percentages of selected articles in each journal reporting a value of prospective power. Black histograms = HIF journals. Gray Histograms = LIF journals. Error bars are 95% CI.

Figure 5 reports the percentages of articles that included a measure of prospective power.

### Use of only NHST without CI, ES, Model or Power estimation

**Figure 6:**
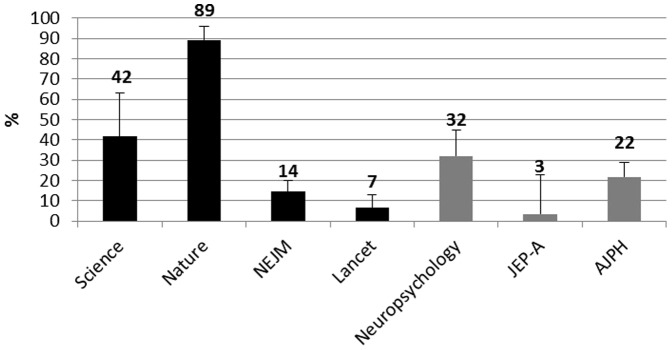
Percentages of selected articles in each journal that used NHST without CI, ES or Model and Power estimation. Black histograms = HIF journals. Gray Histograms = LIF journals. Error bars are 95% CI.

Figure 6 reports the percentages of articles using NHST without CI, ES or Model and Power estimation for HIF and LIF journals with 95% CI.

### CI and ES interpretation

Data related to CI and ES interpretation are reported in the Figure S1 and S2 respectively in the Supplementary Material. They show that the percentages of articles discussing explicitly CI are very low. We observed a maximum of 25% of those articles that reported a CI also including CI interpretation in The Lancet, followed by a 22% in JEP-A, a 14% in Neuropsychology and 3% or 0% in all other journals. The number of articles with ES interpretation is higher with a maximum of 75% of those articles that reported an ES also including ES interpretation in Science—although it is important to note that ES was reported in only 4 articles followed by a 48% in Neuropsychology, 30% in JEP-A, 23% in Lancet, 12% in NEJM and a minimum of 0% in the Nature journals.

### Error bars in Figures

Table S3 in the Supplementary Materials suggests that use of error bars in Figures differed greatly among the journals, probably following explicit or implicit conventions. For example, the prevalent type of error bars reported in Science and Nature journals is the standard error. On the contrary, CIs are mainly reported in NEJM and Lancet. It is interesting to observe the high percentage, 77.8% of figures without error bars in the AJPH.

## Discussion

As to the main focus of this survey, the frequency of the use of NHST without CI, ES or Model and Power estimation among all journals, is quite clear. In the HIF journals this practice (that does not include any of those four techniques) is used in 89% of articles published in Nature, in 42% of articles published in Science whereas it is used only in 14% and 7% of articles published in NEJM and The Lancet respectively. In the LIF journals, this restrictive NHST use ranges from a minimum of 7% of articles in the JEP-A, to a maximum of 32% in Neuropsychology.

The estimation of prospective statistical power in HIF journals ranges from 0% in Science to 66% in The Lancet, whereas in LIF journals, it ranges from 1% of articles published in the AJPH to 23% of articles published in the JEP-A.

The use of CIs in HIF journals ranges from 9% in the articles published in Nature journals, to 93% in the articles published in The Lancet. In LIF journals, this use ranges from 9% of articles published in Neuropsychology, to 78% of articles published in the AJPH. Furthermore the reporting of ES in the HIF journals ranges from a minimum of 3% in Nature journals to a maximum of 87% in Lancet. In the three journals with LIF, this practice is presented in 61% of articles published in Neuropsychology and the AJPH and in 90% of articles published in JEP-A.

The use of model(s) estimation is most prevalent in the articles published in Science, 6 out 24, 25%, although that sample is very small. In all other HIF and LIF journals, this use ranges from 1% to a maximum of 7%.

To summarize, among the HIF journals, the best reporting practices, the use of CI and ES, were present in more than 80% of articles published in NEJM and Lancet whereas this percentage drops to less than 30% in the articles published in Science and in less than 11% in the articles published in the Nature journals. For Science, it is important to note that 25% of the small number of studied used model(s) estimation procedures.

In the LIF journals, ES was used in at least 60% of articles, whereas the use of CI varied considerably, being used in less than 10% of articles published in Neuropsychology and JEP-A, but in 78% of articles published in the AJPH. From the above results, it seems then clear that there is a very large variation among HIF and among LIF journals in the use of alternatives to NHST, with no clear overall difference between the two sets of journals in our study. This variation may reflect the editorial guidelines and varying customs of the different journals. The impact of specific editorial recommendations on the changes in statistical practices, has been documented by [11],[13],[14].

With respect to previous similar studies, we find that for Nature Medicine the use of CIs and prospective power is higher than that reported by [15], referring to 2004 articles. The use of CIs and prospective power was 0% in 2004, whereas we observed a 2/9, 22% and a 1/9, 11% respectively, in 2011, although numbers of articles were small. The same study examined these practices in NEJM. The use of CIs and prospective power was 67% for CIs and 58% for prospective power in 2004, whereas we observed a rise to an 84% and a 61%, respectively, in 2011.

Fidler *et al.* [14] surveyed the use of CIs in the AJPH in the articles published in 1990 and 2000. They observed that this practice rose from 10% to 54%. Our study found that it increased further to 78% in 2011.

Fritz, Sherndl and Kühberger [16] surveyed the use of CI, ES and power analysis in a large number of psychological journals in the period 1990–2010. Overall they found that approximately 10% used CIs, 38% ESs and only 3% power analysis. Approximately the same percentage of CI reporting was observed by [11] in their survey of statistical practices in samples of articles of different psychological journals in the period 1998–2006. In the two psychological journals that had adopted editorial statistical guidelines and were examined in our study, namely Neuropsychology and JEP-A, these percentages range from 9% to 23% for CIs, 61% to 90% for ESs and from 8% to 23% for prospective power.

However, reporting CIs and ESs does not guarantee that researchers use them in their interpretation of results. Note that we used a very liberal approach in the statistical practices classification for ‘interpretation’—any comment about the CI or ES was considered an interpretation. Many authors reported CIs and/or ESs, but this does not guarantee that they use the CI or ES for interpretation, or even refer to them in the text (see Figures S1 and S2). In many cases they used NHST and based interpretation on NHST, with no interpretive reference to the ESs or CIs that they reported. The lack of interpretation of CIs and ESs means that just observing high percentages of CI and ES reporting may overestimate the impact of statistical reform (14). In other words, it is not sufficient merely to report ESs and CIs—they need to be used as the basis of discussion and interpretation.

We emphasize the importance of caution in generalizing our evidence to other disciplines or journals, even noting that the problem of reforming statistical practices has been raised in other disciplines such as biology [17], environmental science [18] and ecology [19].

Our results suggest that statistical practices vary extremely widely from journal to journal, whether IF is high or relatively lower. This variation suggests that journal editorial policy and perhaps disciplinary custom, for example medicine vs. psychology, may be highly influential on the statistical practices published, which in turn suggests the optimistic conclusion that editorial policy and author guidelines may be effective in achieving improvement in researchers' statistical practices.

To summarize our findings, even if we do not endorse the Ioannidis [20] claim that “most published research findings are false”, we are convinced that without an elimination of the “Null Ritual” and a rapid adoption of a statistical reform, “most published research findings have a high probability of being false”.

## Supporting Information

Table S1 **2011 Social Science Report Impact Factor (IF) for journals included in the study.**
(DOCX)

Table S2 **Number of 2011 articles included for each journal.**
(DOCX)

Table S3 **Types of error bars reported in Figures.**
(DOCX)

Figure S1 **Percentages of articles reporting a CI that included CI interpretation.** Black histograms = HIF journals. Gray Histograms = LIF journals. Error bars are 95% CI.
(TIF)

Figure S2 **Percentages of selected articles in each journal reporting an ES that included ES interpretation.** Black histograms = HIF journals. Gray Histograms = LIF journals. Error bars are 95% CIs.
(TIF)

Coding Manual S1 **Complete scoring method of articles passing the inclusion criteria.**
(DOCX)
