# Supplementary material for: High Impact = High Statistical Standards? Not Necessarily So
Source: PLoS One. 2013 Feb 13;8(2):e56180. doi: 10.1371/journal.pone.0056180 (PMC3571951; doi:10.1371/journal.pone.0056180)
Supplement: Table S1 — 2011 Social Science Report Impact Factor (IF) for journals included in the study. (DOCX) [file pone.0056180.s001.docx]

Table S1: 2011 Social Science Report Impact Factor (IF) for journals included in the study

| Journal | IF |
| --- | --- |
| *The New England Journal of Medicine* | 53.2 |
| *Lancet* | 38.2 |
| *Nature* | 36.2 |
| *Science* | 31.2 |
| *Nature Medicine* | 22.4 |
| *Nature Neuroscience* | 15.5 |
| *American Journal of Public Health* | 3.9 |
| *Neuropsychology* | 3.8 |
| *Journal of Experimental Psychology-Applied* | 2.2 |

*Note*. The top six journals were considered HIF and the bottom three LIF.
