# Supplementary material for: High Impact = High Statistical Standards? Not Necessarily So
Source: PLoS One. 2013 Feb 13;8(2):e56180. doi: 10.1371/journal.pone.0056180 (PMC3571951; doi:10.1371/journal.pone.0056180)
Supplement: Table S2 — Number of 2011 articles included for each journal. (DOCX) [file pone.0056180.s002.docx]

Table S2: Number of 2011 articles included for each journal

| Journal | *N* |
| --- | --- |
| *The New England Journal of Medicine* | 173 |
| *Lancet* | 122 |
| *Nature* | 5 |
| *Nature Medicine* | 9 |
| *Nature Neuroscience* | 23 |
| *Science* | 24 |
| *American Journal of Public Health* | 147 |
| *Neuropsychology* | 75 |
| *Journal of Experimental Psychology-Applied* | 30 |
