# Supplementary material for: High Impact = High Statistical Standards? Not Necessarily So
Source: PLoS One. 2013 Feb 13;8(2):e56180. doi: 10.1371/journal.pone.0056180 (PMC3571951; doi:10.1371/journal.pone.0056180)
Supplement: Table S3 — Types of error bars reported in Figures. (DOCX) [file pone.0056180.s003.docx]

Table S3: Types of error bars reported in Figures.

|  | **SD** | **SE** | **CI** | **Others** | **Error bars not included** |
| --- | --- | --- | --- | --- | --- |
| Science | 0 | 100 | 0 | 0 | 0 |
| Nature | 2.7 | 94.6 | 2.7 | 0 | 0 |
| NEJM | 4.4 | 11.8 | 50.7 | 7.4 | 25.7 |
| Lancet | 4.3 | 7.6 | 65.2 | 6.5 | 16.3 |
| Neuropsychology | 5.8 | 40.4 | 3.8 | 44.2 | 5.8 |
| JEP-A | 0 | 70 | 15 | 0 | 15 |
| AJPH | 2.2 | 8.9 | 8.9 | 2.2 | 77.8 |

SD = Standard deviation; SE = Standard error; CI = Confidence Interval;

Others = e.g. box-plots;
