# Supplementary material for: High Impact = High Statistical Standards? Not Necessarily So
Source: PLoS One. 2013 Feb 13;8(2):e56180. doi: 10.1371/journal.pone.0056180 (PMC3571951; doi:10.1371/journal.pone.0056180)
Supplement: Coding Manual S1 — Complete scoring method of articles passing the inclusion criteria. (DOCX) [file pone.0056180.s006.docx]

**Coding Manual**

**General Instructions**

We are coding empirical articles related to human participants related to psychology or clinical medicine, reporting new data only. We are not coding: theoretical, review articles or meta-analyses, single-case, in vitro, animal studies.

We are searching the abstract, method, results and discussion sections of papers for the items below.

We are coding the systematic reporting (always used when necessary) of each of the items below.

1. **NHST reported?**

For this item, we will code whether NHST was reported in either the text or a table and/or whether it was displayed in a figure.

Any instance of one of the following, in either of the above formats, is sufficient for considering NHST reported:

- *p* value
- Asterisks and footnotes (e.g.*)
- “statistically significant” or “statistically non-significant” (but not the word ‘significant’ alone)
- the abbreviation *ns*
- α=.05 (or .01 etc)

**Attention**: First check if there are CIs, ESs, Model and Power estimation measures. If none of these measures is detected, consider NHST, and include the article in the NHST category if it includes any of the items listed above.

1. **CI reported?**

As for item 1, we will code whether CIs (e.g. 90%, 95%) were reported in either the text or a table and/or whether they were displayed in a figure.

- In text or tables CIs may appear as: (3.2, 4.4) OR (3.8 ± 0.6) OR (3.2 to 4.4) etc, or may be a mention in words without numbers, e.g. “the confidence interval on the median did not include the predicted value”.
- In a figures they may appear as error bars, or as extra lines or curves flanking a regression line or curve

Use a lenient criterion: reported for at least one measure

**3. Interpretation: CI (text, table or figure) & SE (bars in figure only)**

We code whether *any* CI is interpreted, regardless of whether it appears in text, table or figure.

We also code interpretation of SEs but only when SEs appear as bars in figures.

Coding of interpretation is lenient, that is, we code as interpretation **any** statement beyond mention of where the CI or SE appears (e.g. “Figure 2 shows means with 95%CIs” is not interpretation).

Common types of interpretation might include:

- any mention of width, length or extent of the CI or SE bar(e.g. “Note however that the CI is wide”, “The extent of CI suggests…”, etc)
- any mention of overlap, or lack of overlap, of CIs or SE bar (e.g. “There is no overlap between the CIs for …”)
- any mention of the CI or SE bar including the null or some other particular value (e.g. “The CI covers zero…”, “The CI includes the threshold value…”, “The CI includes the cut off value…”)
- any use of the word ‘precision’ in association with a CI or SE bar. This will (probably) most often be used in conjunction with a statement about the width, length, or extent of the interval, but it may also occur separately.

**4. ES without CI is reported?**

Effect size is reported in either the text or a table. It may be reported as standardized measures (d, r, odds ratio) or as raw measures but in this case it may be explicated that they refer to effect size.

Use a lenient criterion: reported for at least one measure

1. **ES with CI is reported?**

Effect size with corresponding CIs is reported in either the text or a table

Use a lenient criterion: reported for at least one measure

1. **ES is interpreted?**

In the Comments or Discussion sections there is at least an explicit statement of ESs measures as qualifications of results.

1. **Prospective Power reported?**

Prospective statistical power is used in the study planning and/or is estimated in the results section.

1. **Models comparison or estimation is reported**

When NHST is reported with or substituted with models comparison or estimation techniques (e.g. Bayesian, AIC, structural equation modeling, etc.)

1. **Error bars reported in Figures? Of which type (SD, ES, CI, etc., not reported)?**

Use a lenient criterion: reported for at least one measure.
